# Does alveolar ridge preservation reduce the need for sinus floor elevation: A comparative study to spontaneous healing

**DOI:** 10.1111/cid.13391

**Published:** 2024-09-26

**Authors:** Elias Jean‐Jacques Khoury, Keyvan Sagheb, Bilal Al‐Nawas, Jochem König, Eik Schiegnitz

**Affiliations:** ^1^ Department of Oral and Maxillofacial Surgery University Medical Center Mainz Mainz Germany; ^2^ Institute for Medical Statistics, Epidemiology and Informatics University Medical Center of the Johannes‐Gutenberg‐University Mainz Mainz Germany

**Keywords:** alveolar ridge preservation, ARP, bone substitute, implants, prospective clinical study, sinus floor elevation, socket healing

## Abstract

**Introduction:**

In cases of atrophy in the maxillary posterior region, characterized by reduced vertical bone volume, implant placement becomes challenging. Augmentation procedures like sinus lifts are often needed to address insufficient bone volume. This study aims to explore if alveolar ridge preservation, using a bovine bone substitute and a porcine collagen membrane, significantly decreases the need for sinus lifts compared to natural wound healing after tooth extraction.

**Materials and methods:**

In this comparative clinical study, 40 patients requiring a total of 53 extractions were assigned to one of the following groups: a test group with bovine bone substitute material (Straumann® XenoFlex) and a porcine collagen membrane (Jason® membrane), or a control group with spontaneous socket healing. After 6 months, digital volume tomography was performed for implant planning.

**Results:**

For seven patients from the control group (*n* = 22 extracted sites) sinus lift augmentations were performed while only four sinus lift procedures were performed in the test group (*n* = 31 extracted sites), indicating a higher need for sinus augmentation procedures in the control group, however not statistically different on a *p* value of 0.05 (*p* = 0.168). In the control group, the mean value of the radiographically measured bone height (mesial and distal) was 11.13 ± 2.12 mm preoperatively before tooth extraction, while it was 11.3 ± 2.17 mm postoperatively after implant placement. In contrast, the mean value in the test group was 11.78 ± 3.09 mm preoperatively and 11.92 ± 2.79 mm postoperatively. Statistical analysis revealed no significant difference between the two groups (odds ratio 0.32; 95% CI: 0.08, 1.26; *p* = 0.951). The implant survival rate in the control group was 100%, compared to 96.77% in the test group.

**Conclusion:**

Within the limits of this study, the use of bovine bone substitute and a porcine resorbable membrane after tooth extraction in the posterior maxilla seems to reduce the need for sinus augmentation in comparison to spontaneous healing although the difference was not statistically significant. Additionally, the Alveolar Ridge Preservation in the test group made external sinus floor elevation unnecessary compared to the control group. The change in radiographically measured bone height pre‐ and postoperatively showed no significant difference between the two groups.

**Summary box**

Atrophy of the alveolar ridge after tooth extraction in the posterior maxillary region presents a significant challenge for implant placement. Systematic reviews have shown that Alveolar ridge preservation can effectively reduce the dimensional decrease of the alveolar ridge typically occurring after tooth extraction, thereby reducing the need for further augmentations.

This study is a prognostic study investigating whether alveolar ridge preservation can reduce the need for sinus floor elevation in the posterior maxilla.

## 1 INTRODUCTION

Numerous studies and reviews have shown that tooth extractions cause changes in alveolar bone morphology with structural and dimensional adjustments.^1^, ^2^, ^3^, ^4^ Bone resorptions were observed 6 months after tooth removal, with horizontal dimensional reduction of 29%–63% and vertical dimensional reduction of 11%–22%,^5^ while two‐thirds of the reductions occurred within the first 3 months.^6^ After tooth extraction, the bundle bone that lines the periodontal ligament around the tooth and anchors the sharpey fibers in the bone is resorbed^6^ and replaced with woven bone, causing a vertical reduction of the alveolar crest.^7^ Subsequently reabsorption occurs from the outer surfaces of both bone walls.^7^ Additionally Araujo and colleagues reported a larger ridge reduction in the molar than in the front region.^1^

Furthermore, a process called pneumatization takes place in the maxillary sinus. It describes a physiological process of the maxillary sinus characterized by the expansion of the sinus over time.^8^ Several factors have been linked to its occurrence, including bone density, respiratory air pressure within the sinus in combination with extraction of posterior teeth, heredity, and previous sinus surgery.^7^ It is likely that the sinus membrane will shift more coronally and occupy the space of the extraction socket if there is no or only a thin bundle bone between the sinus and the root apex as the extraction socket cannot resist the pressure from the sinus.^8^, ^9^

Depending upon individual local and systematic factors, the degree of bone remodeling may vary but generally results in both horizontal and vertical irreversible alveolar ridge reduction.^10^

The loss of bone and the susceptibility of the maxillary sinus to pneumatization after a tooth extraction may limit the subsequent placement of an implant and the preservation of the bony structures is a prerequisite for a functional and aesthetic result.^8^ Implants are advised to be provided with bone walls about 1‐ to 2‐mm wide on buccal and lingual aspects in order to maintain a stable bone height.^1^ Thus, many authors suggest preserving the alveolar ridge by using various bone grafts, such as bovine‐derived xenografts, autologous bone, allografts, and alloplasts which promote hard tissue formation or proposed the use of different types of implant placement.^7^, ^11^ A variety of successful procedures have been reported for maintaining the anatomical dimensions of the alveolar ridge and previous data indicated that alveolar ridge preservation (ARP) could prevent the pneumatization process and therefore reduce the need for subsequent sinus augmentation.^8^, ^10^, ^12^

Some authors demonstrated that compared to non‐grafted sites, the profile of ridges treated by means of ridge preservation is better preserved than those without grafts and previous systematic reviews have shown solid evidence supporting their success.^10^, ^11^, ^13^ However, the treatment could neither completely prevent volume nor the contour alterations.^13^

The aim of this study was to investigate the influence of the alveolar ridge preservation after tooth extraction in the posterior maxilla with a bovine bone substitute and a collagen membrane on the need for sinus lift augmentation compared to spontaneous healing. The primary outcome criterion is the avoidance of an internal or external sinus lift. Secondarily, the vertical bone loss in the area of the extraction socket was measured. Furthermore, the success rate of the inserter implant in this study is investigated.

## 2 MATERIALS AND METHODS

### 2.1 Study design and population

**FIGURE 1:**
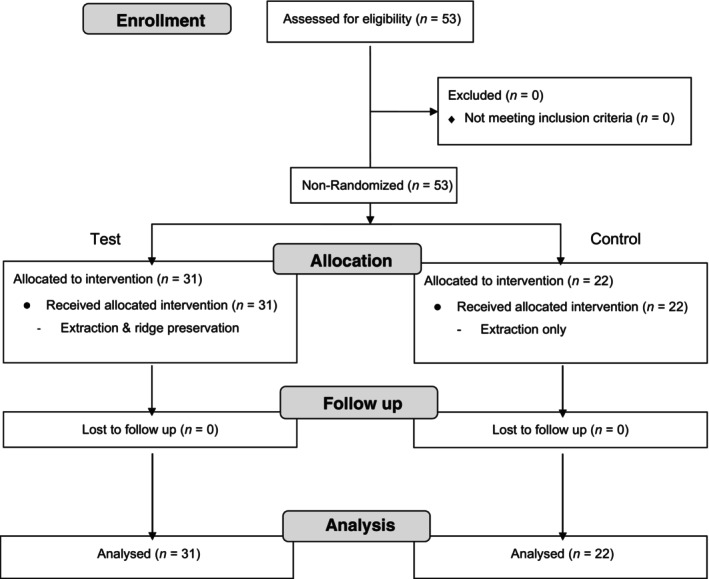
CONSORT flowchart of the study.

This study was designed as a prospective non‐randomized controlled clinical trial with two parallel groups. The study was approved by the ethical commission relevant state medical chambers (No. 202‐15160). The CONSORT flowchart for non‐randomized controlled clinical trial is presented in Figure 1.^14^

This study was performed from July 8, 2021 to January 22, 2024. All the patients were older than 18 years and provided informed consent for the surgery. Patients were recruited from the patient pool of the Department of Oral and Maxillofacial surgery—plastic surgery of the University. The study protocol was approved by the Ethics Committee of the University Medical Centre under the number: 2020‐15160.

Patients with posterior teeth or roots in the posterior maxilla candidate to extraction were allocated into two groups. Allocation was performed by assigning the first 22 patients, with a total of 31 teeth to be extracted, to the test group, while the subsequent 18 patients were assigned to the control group. Allocation was performed by assigning the first 22 patients, with a total of 31 teeth to be extracted, to the test group, while the subsequent 18 patients, with a total of 22 teeth to be extracted, were assigned to the control group. In some patients, more than one tooth was extracted, but no more than three teeth were extracted from a patient. An implant was placed in every region where a tooth was removed. The study was designed as a controlled clinical trial. Patients of test group were treated by alveolar ridge preservation using bovine bone grafting material (Straumann® XenoFlex) and porcine collagen membrane (Straumann® Jason® membrane). In patients of the control group, no grafting was performed. After 6 months of healing Cone‐beam computed tomography's (CBCT) were taken, for implant planning.

The necessity of sinus floor elevation between the test and control group was determined depending on the extent of available vertical bone before implant placement (treatment modalities for implant placement) and changes in vertical bone dimensions were measured radiologically and compared at baseline and after implant surgery. Implant survival, success rates, and radiographic changes at the crestal bone level by peri‐apical x‐ray and panoramic tomography were evaluated.

### 2.2 Inclusion criteria

The following inclusion criteria were applied:

- Patients aged ≥18 years.
- Patients requiring extraction of maxillary posterior teeth (molars, first premolars and second premolars).
- Patients willing to participate in this study and no systematic or local conditions that would preclude them from implant therapy.
- Patients who were healthy.
- No systematic or local conditions presenting a contraindication to extraction and implant placement.

### 2.3 Exclusion criteria

The following exclusion criteria were applied:

- General contraindication to implant surgery.
- Extraction of more than three adjacent teeth.
- Pregnant or nursing women.
- Uncontrolled diabetes.
- History of oromaxillofacial radiation therapy.
- Smoking.
- Osteogenesis‐related diseases or medications affecting bone formation.
- Pathologic conditions of the maxillary sinus.
- Untreated severe periodontitis with poor oral hygiene.
- Teeth with symptoms of an acute infection such as tapping sensitivity or in conjunction with an abscess.

### 2.4 Handling of correlated data

Among 40 patients, 53 posterior teeth were extracted. The test group included 22 patients with a total of 31 teeth to be extracted, while the control group consisted of 18 patients with 22 teeth to be extracted.

### 2.5 Treatment modalities for implant placement

Implants with a diameter of 3.5, 3.75, 4, and 4.5 mm and length of 6, 8, 10, and 12 mm were used depending on the bone level. The treatment strategies for implant surgery and the need for sinus lifts were prepared according to the residual bone height (RBH), assessed from CBCT images taken 6 months after extraction. The following protocol was used for further patient management:

- ≥6 mm residual vertical bone height: placement of Straumann® BLX Implant
- 3–5 mm residual vertical bone height: internal sinus lift with simultaneous implant insertion
- 0–3 mm residual vertical bone height: external sinus lift with two‐staged implant insertion after 4 months

### 2.6 Treatment procedure

#### 2.6.1 Tooth extraction

**FIGURE 2:**
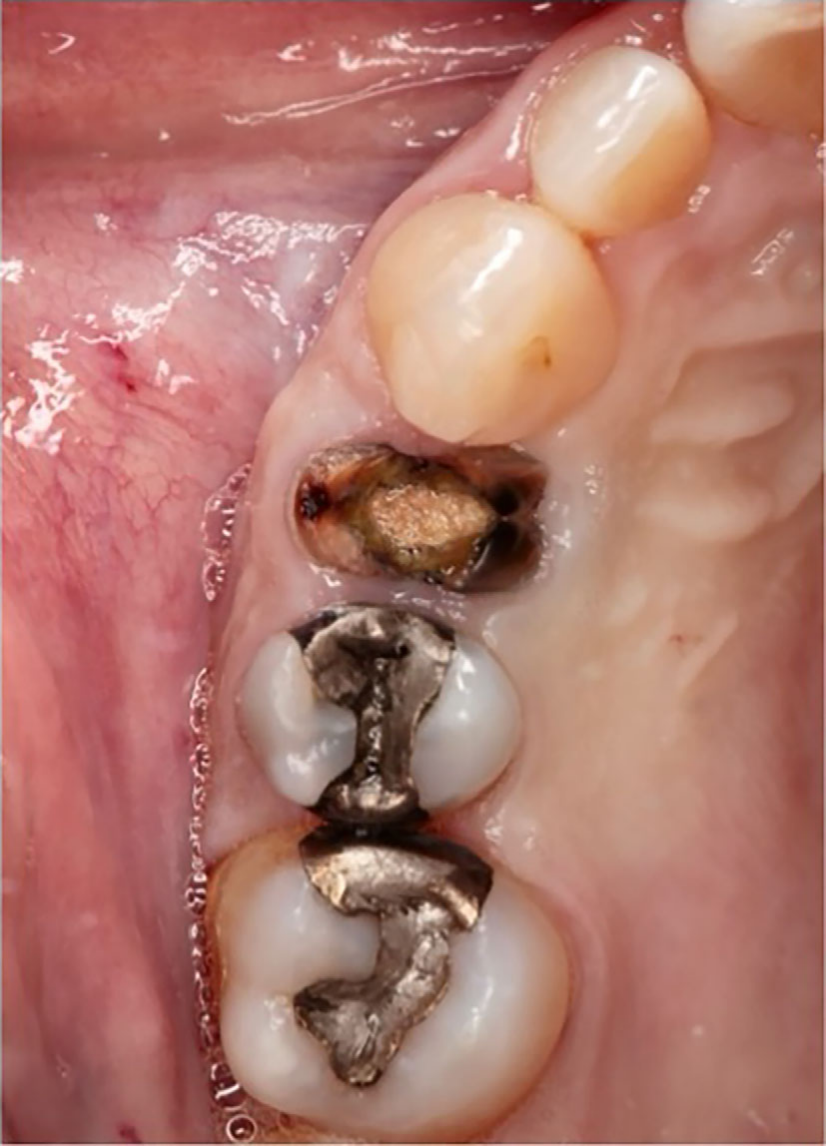
Tooth 14 not worth preserving.

**FIGURE 3:**
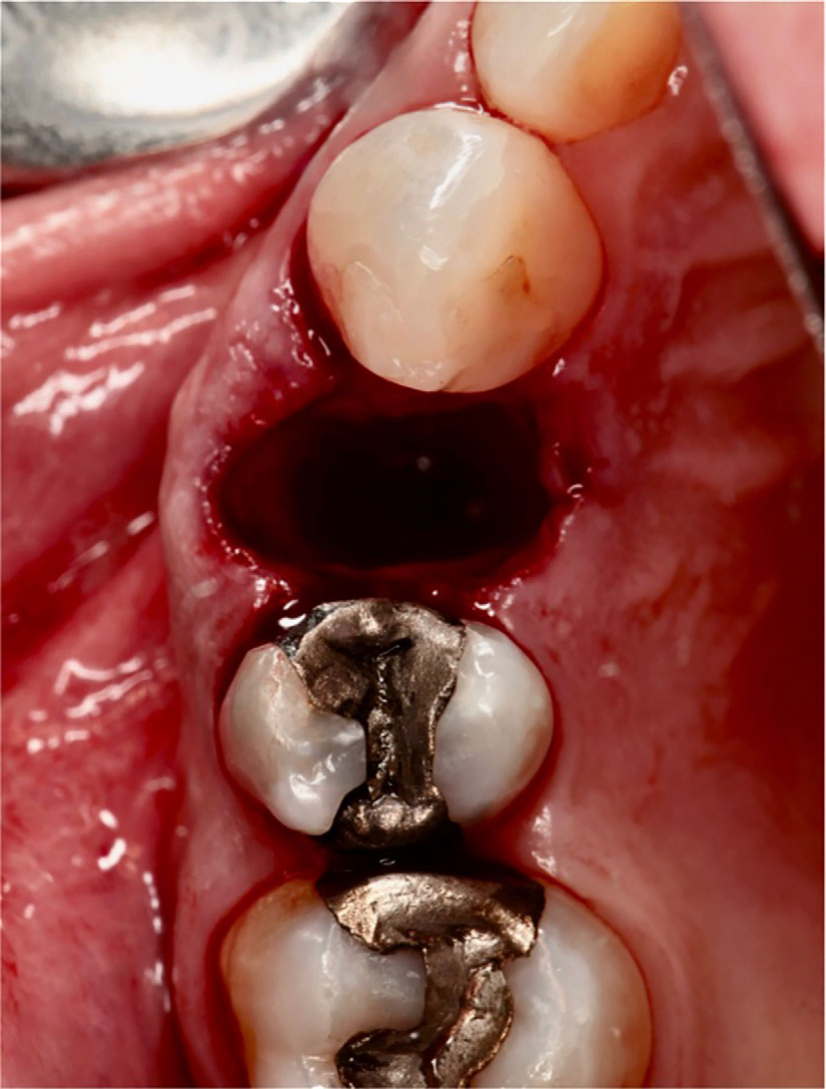
Condition after atraumatic tooth extraction.

Extractions of the tooth which had to be removed were performed as atraumatically as possible using periotomes, elevators, and extraction force. After tooth extraction, the socket was carefully curetted, and all granulation tissue was removed (Figures 2, 3, 8, and 9).

If the patient belonged to the control group, the wound edges of the alveolus were adapted after sufficient coagulation with the help of adaptation sutures using non‐resorbable suture material Ethilon™ (5‐0, Ethicon®, Germany).

**FIGURE 4:**
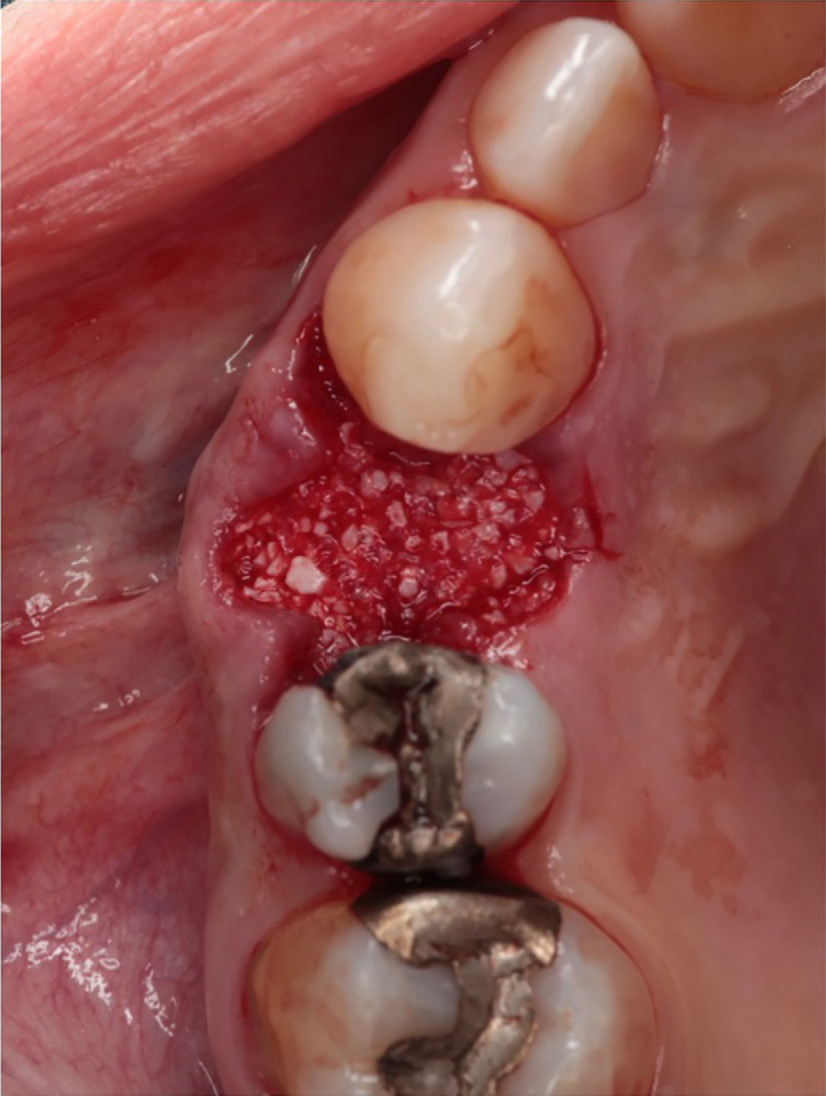
Filling the extraction socket with bovine bone substitute material.

**FIGURE 5:**
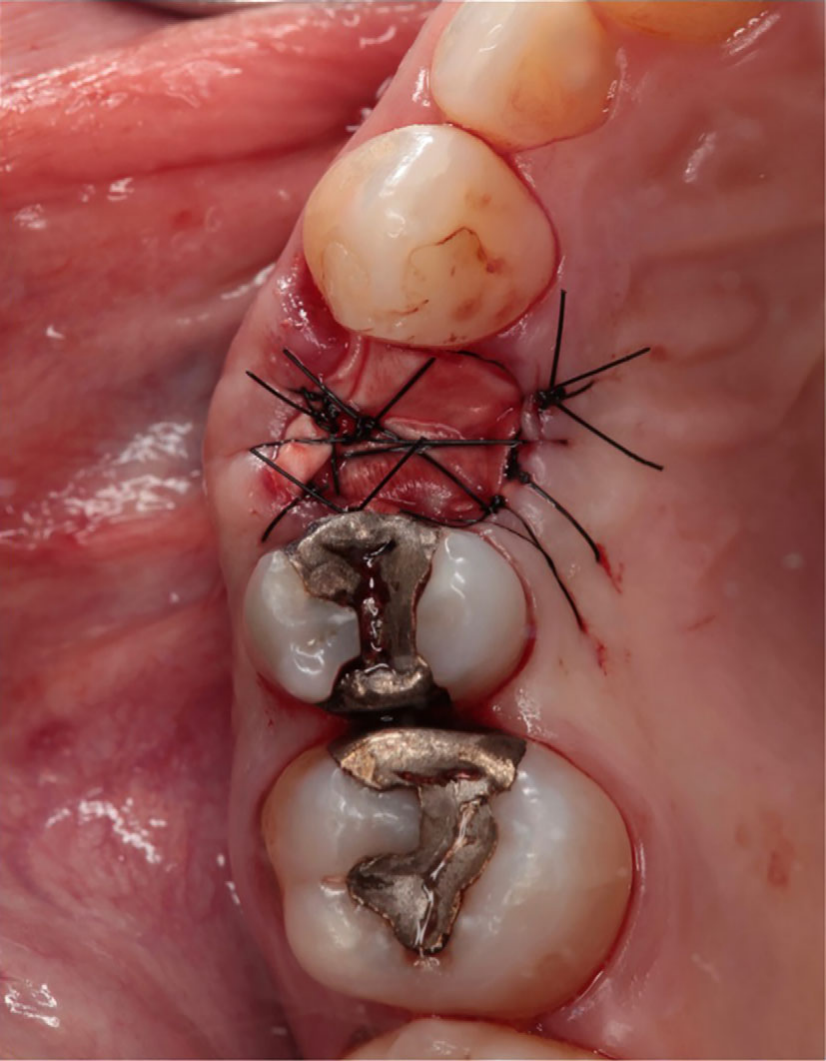
Suture after application of a collagen membrane.

In the test group, the bovine bone graft substitute was placed directly into the alveolus using an applicator and covered with a resorbable membrane (Figures 4 and 5). Perioperatively, the patients in the test group received an oral administration of prophylactic antibiotic therapy of 2.0 g Amoxicillin/Clavulanic Acid (875 mg/125 mg) 1 h before surgery. No attempt was made to augment the ridge vertically above the height of the crest. The wound margins were covered over the membrane with non‐resorbable suture material Ethilon™ (5‐0, Ethicon®, Germany). Primary wound closure was not attempted. For both groups, Valsalva maneuver was performed to check whether Schneiderian membrane was damaged. A recall appointment was scheduled 7–14 days after the surgery for suture removal. The membrane barrier was left in place in all cases.

#### 2.6.2 Evaluations after the first surgery (tooth extraction)

All patients were recalled evaluating the wound healing. A CBCT was conducted 6 month‐post‐operatively for the purpose of planning implant placement.

#### 2.6.3 Implant insertion

**FIGURE 6:**
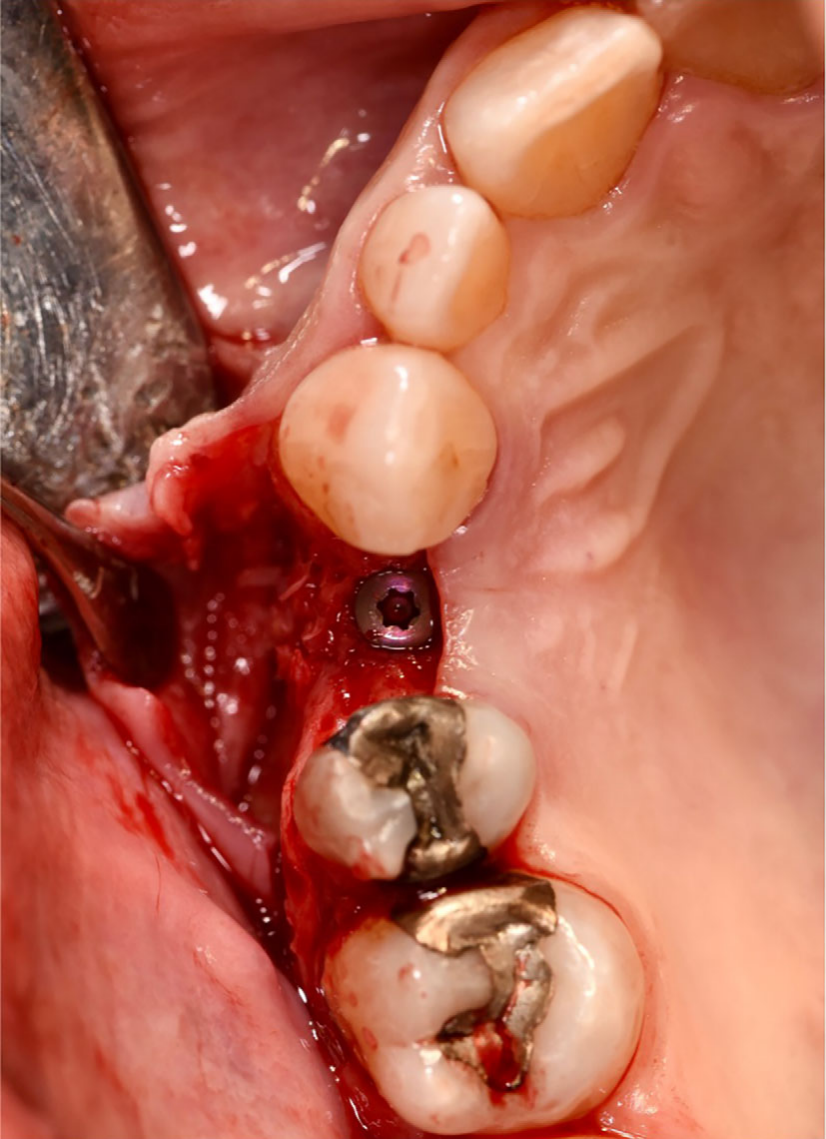
Implant placement.

All patients received an oral administration of prophylactic antibiotic therapy of 2.0 g Amoxicillin/Clavulanic Acid (875 mg/125 mg) 1 h before surgery. In case of allergy, Clindamycin 600 mg 1 h before surgery was given. Individualized oral hygiene instructions were provided prior to any surgical intervention and patients had to rinse with 0.2% chlorhexidine solution for 1 min. Conventional crestal incisions were applied under local anesthesia (Ultracain® adrenaline 1:200,000, Septodont, Niederkassel, Germany), followed by the reflect of full thickness soft tissue flap in healed ridge. Sequential osteotomy and implant insertion is performed according to the manufacture's guidelines (Straumann® BLX, Institut Straumann AG, Switzerland) (Figures 6, 10, 11, and 12).

**FIGURE 7:**
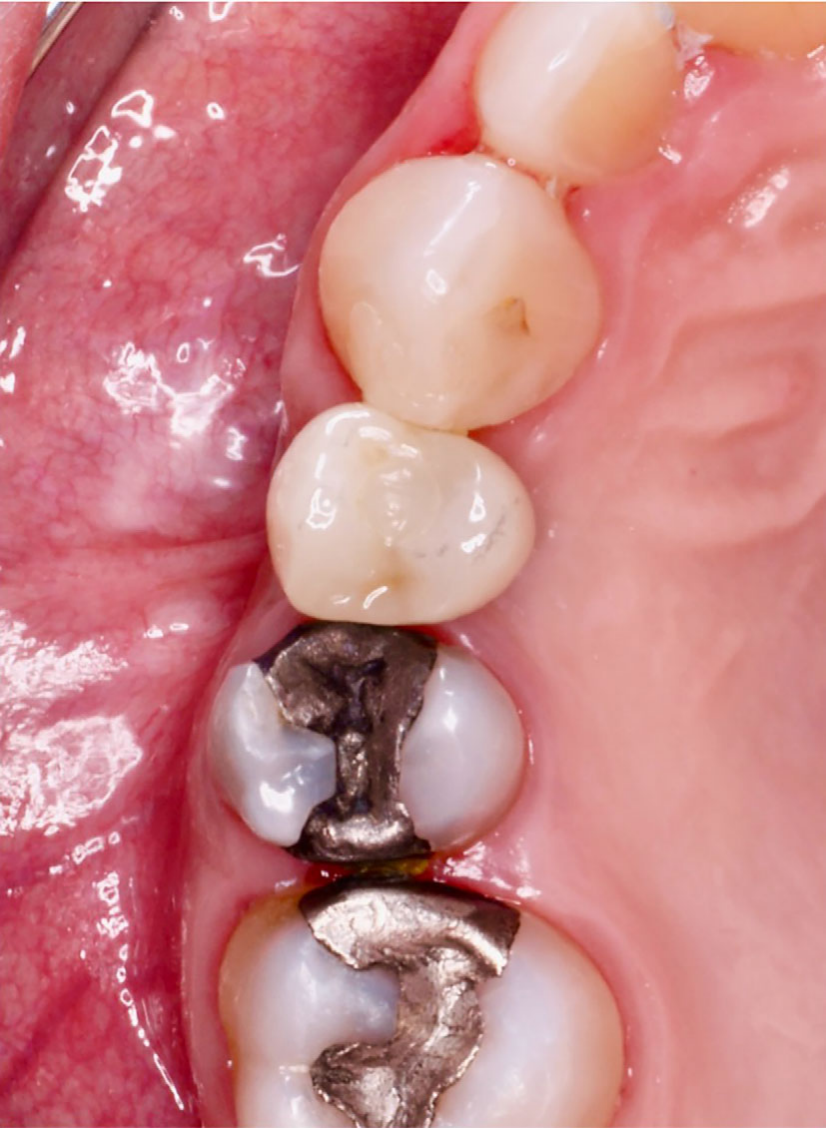
Prosthetic restauration of the implant.

In case of insufficient vertical bone height as shown in the treatment modalities for implant placement, a sinus lift procedure was performed. Mid crestal incision was made with mesial vertical releasing incision. The internal sinus floor elevation began with the crestal incision and the preparation of the soft tissue. The crestal approach was performed through the pilot hole until just before the Schneiderian membrane. By using condensers and osteotomes, which contribute both to the compaction of the bone and to the targeted perforation and elevation of the sinus floor, the sinus floor could be elevated by a few millimeters so that the implant could be inserted at the desired length. For the external sinus floor elevation, a small window was created on the side of the maxillary sinus using a round diamond bur under copious saline irrigation. The Schneiderian membrane was carefully elevated using different sinus elevation curettes. Following complete elevation of the membrane, the sinus cavity was augmented by bovine bone grafting material which was mixed with saline taking care not to perforate the sinus membrane during packing of the graft. Wound closure was achieved using resorbable suture Vicryl™ (4‐0, Ethicon®, Norderstedt, Germany). All implants were prosthetically restored (Figures 7 and 13). Follow‐up of the restored implant took place 1 year postoperatively.

##### Patient from the test group

##### Patient from the control group

**FIGURE 8:**
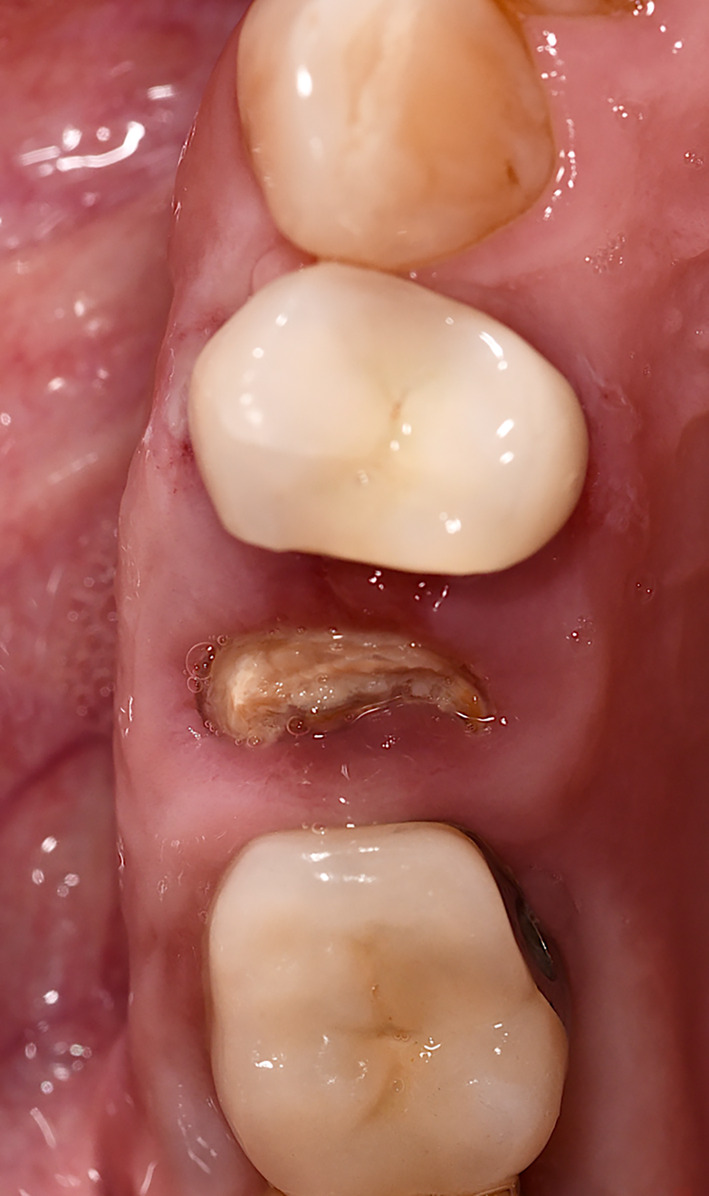
Tooth 15 not worth preserving.

**FIGURE 9:**
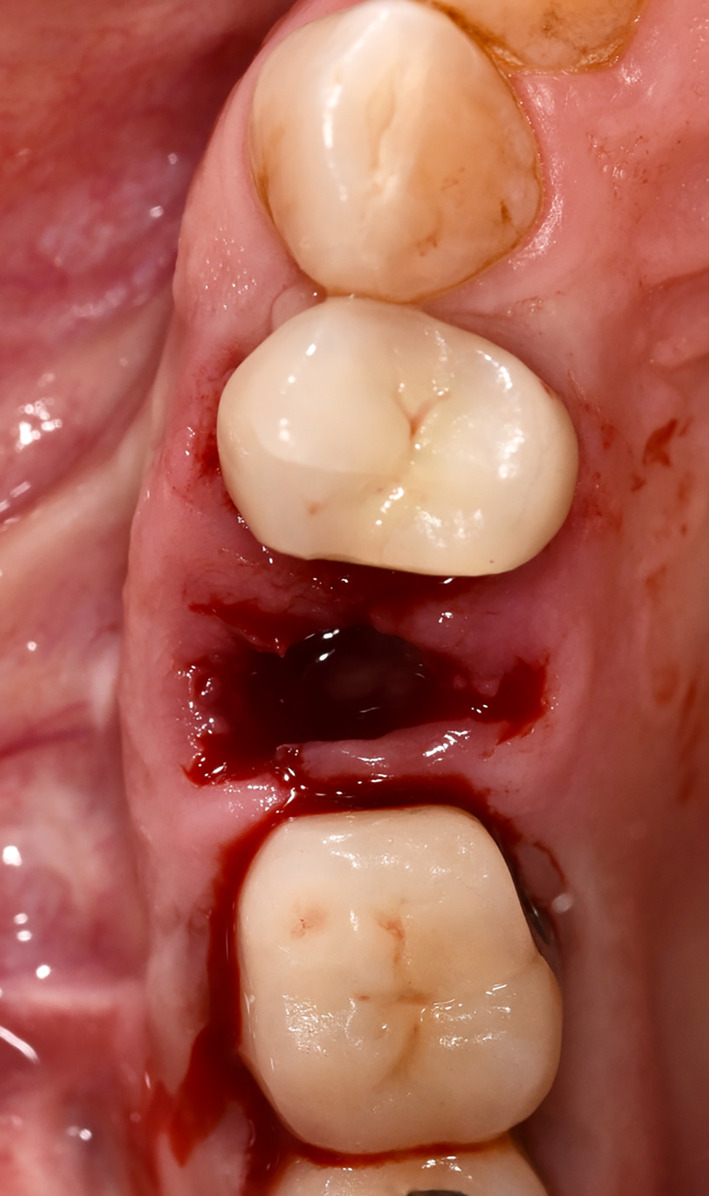
Condition after atraumatic tooth extraction.

**FIGURE 10:**
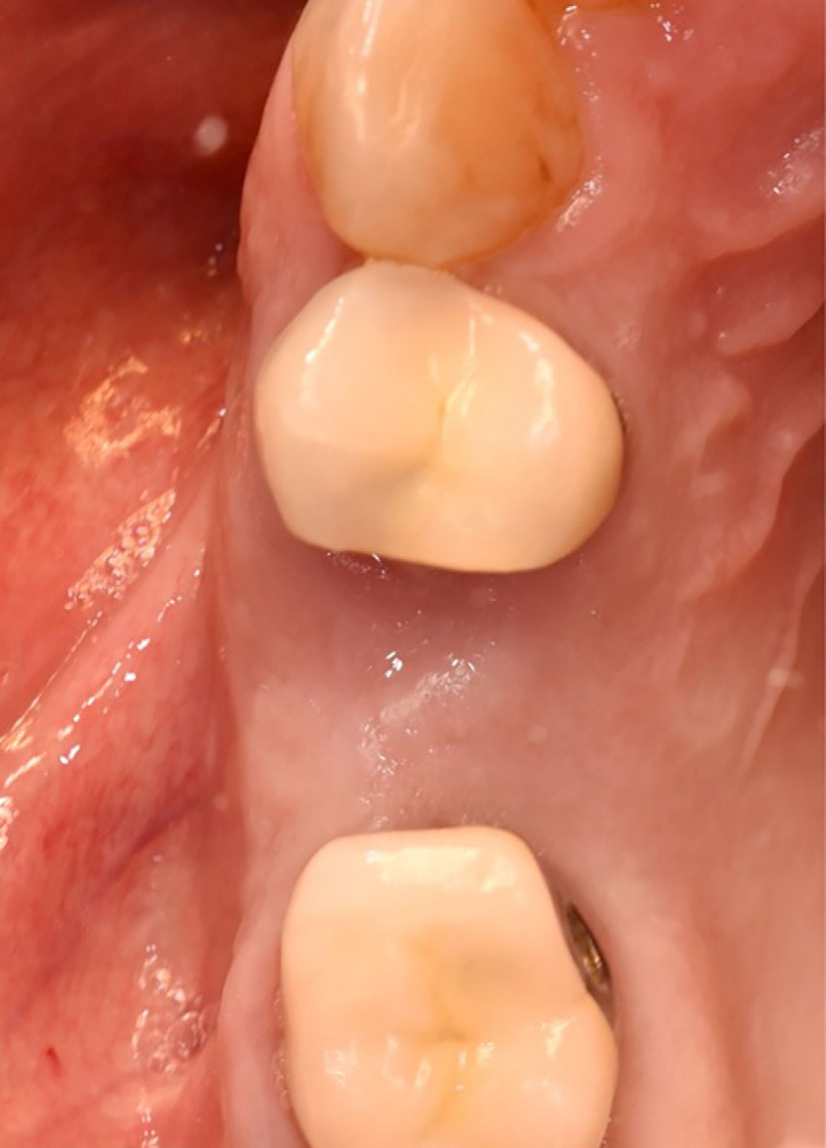
Occlusal view after 6 months.

**FIGURE 11:**
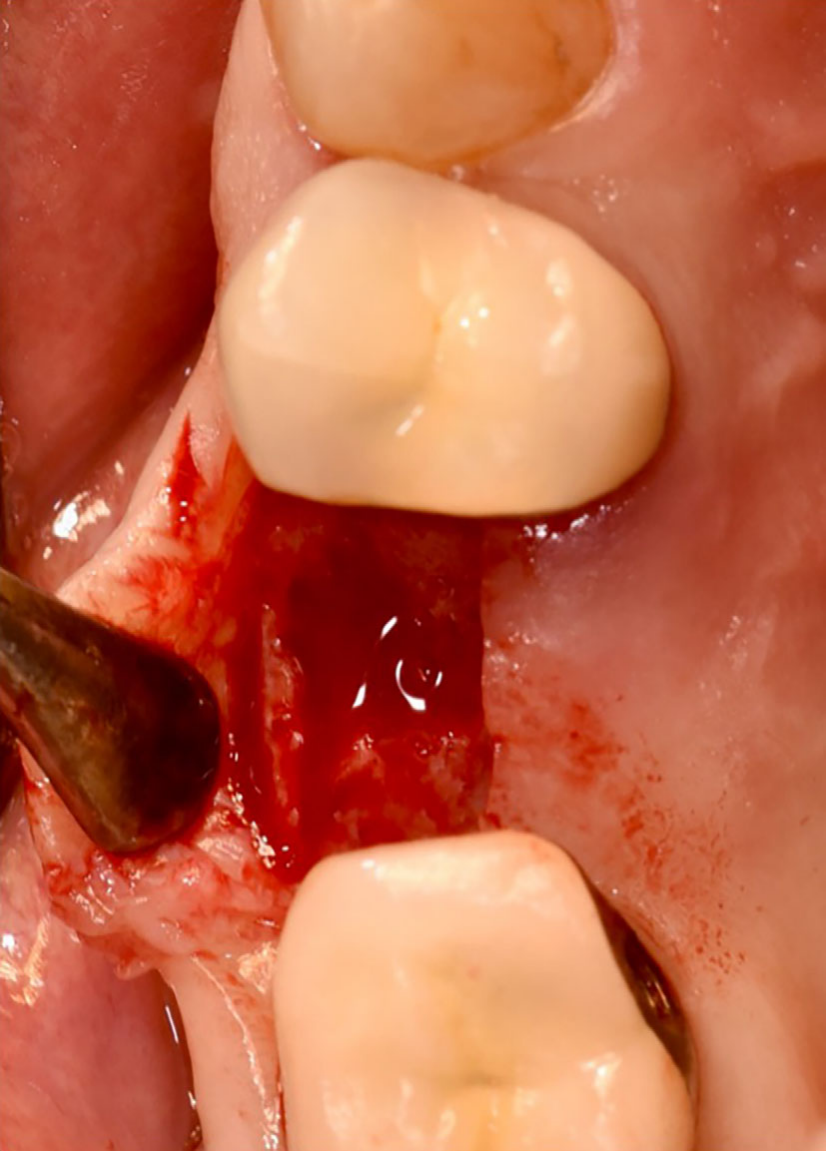
Occlusal view of alveolar ridge at surgical reentry.

**FIGURE 12:**
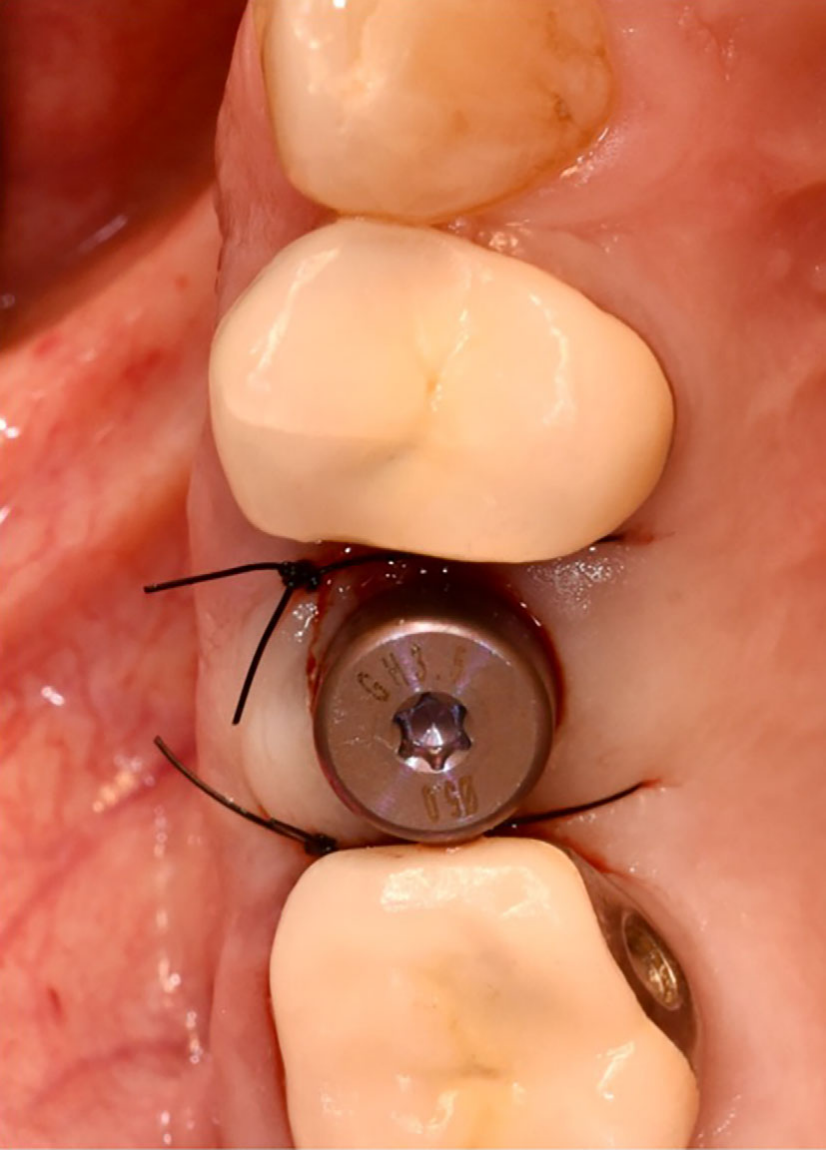
Implant placement with the healing abutment.

**FIGURE 13:**
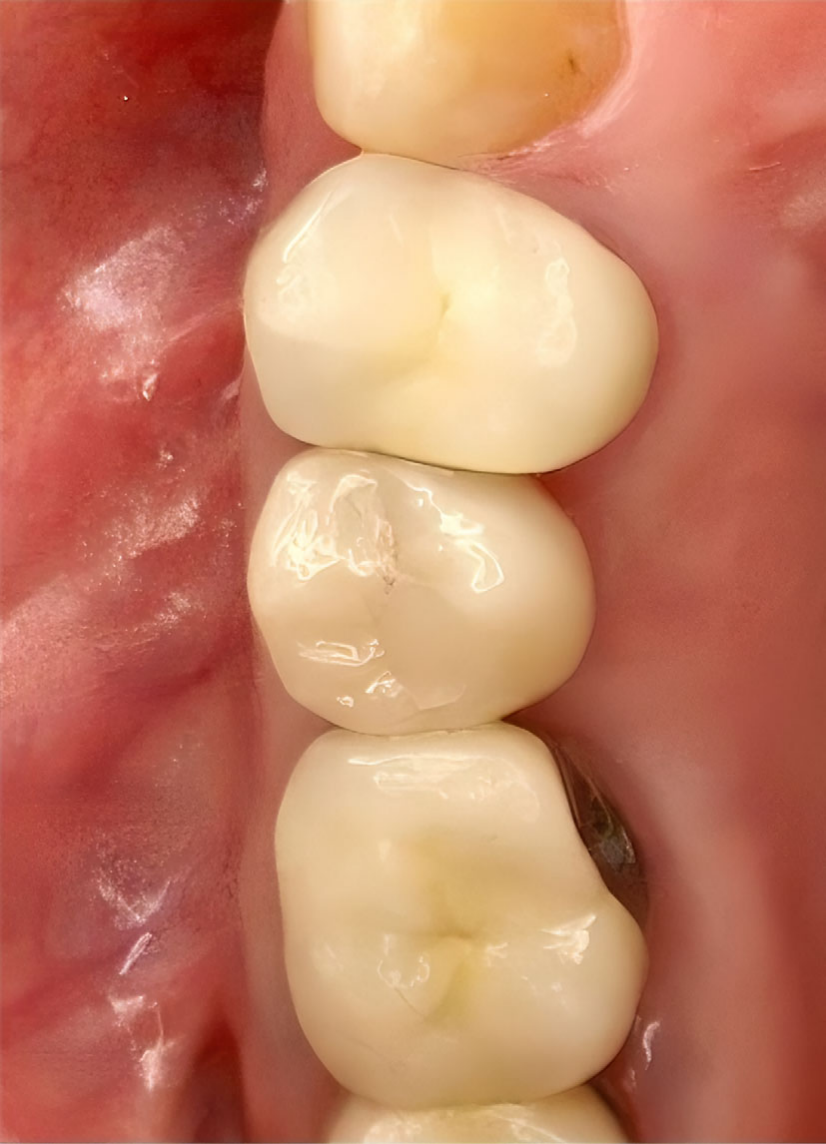
Prosthetic restauration of the implant.

## 3 OUTCOME MEASUREMENTS

### 3.1 Radiographic analysis

To evaluate the vertical bone height, x‐ray images were examined. However, this examination is limited due to the low resolution and the fact that the 3‐dimensional event is reduced to a 2‐dimensional image. For the presented study, both panoramic slice images and intraoral dental radiographs were taken and evaluated. Compared to the intraoral dental radiographs, the panoramic radiograph has a lower level of detail reproduction. Due to the known implant dimension, it was possible to correct the measured values. For this purpose, the implant length shown from the abutment to the apex was measured on the computer and compared with the length specified by the manufacturer and adjusted. The vertical bone height was then measured according to this standard, which made it possible to correct the typical image distortion errors. The radiograph taken before the tooth was extracted was used as the basis for the comparison and the bone level was determined mesially and distally of the tooth. The radiograph directly after insertion of the implant was then used as the comparison image and the bone level was determined mesially and distally of the implant. The mean values of the mesial and distal vertical bone height were then determined preoperatively and postoperatively and compared with each other to assess whether the bone height had increased, decreased, or remained the same. It is to be noted that for the present study, only vertical height changes could be analyzed.

Because some cases (*n* = 11) underwent sinus floor elevations prior or in combination to implant placement and thus gained new vertical bone height, these cases were excluded from the analysis of the radiographic examination to determine bone resorption. Thus, the analysis focused exclusively on those cases who did not require further augmentation procedures.

All measurements were performed by the same single, experienced investigator.

### 3.2 Success criteria of the inserted implant

In 1990, the success criteria, which adhered to the definition outlined by Buser and colleagues,^15^ were described as follows:

- The implant is in situ.
- No mobility of the implant.
- No peri‐implant infection with putrid secretion.
- No persistent peri‐implant radiotranslucency.
- No persistent discomfort such as pain, foreign body sensation, or dyesthesia.

### 3.3 Statistical analysis

The statistical analyses were performed using the SPSS program, version 27 (IBM®, USA) for the statistical analysis of the data. The study is descriptive and exploratory in nature with the primary initial hypothesis that ridge preservation prevents the need for sinus floor elevation. Means, standard deviations and confidence intervals were calculated for each factor. Descriptive statistical analysis involved determining mean values and standard deviations for continuous variables and evaluating frequency distributions for categorical variables. Normality of continuous variables was checked using Shapiro–Wilk tests. *p*‐Values were derived using *t*‐tests or Mann–Whitney *U* test for continuous variables that followed an apparently normal distribution, Mann–Whitney *U* tests for continuous variables not normally distributed, and Chi‐square tests with Fischer's exact test for categorical variables. All *p*‐values were two‐sided. Box plots and bar charts were created to graphically illustrate differences. The alpha error was set at 0.05.

## 4 RESULTS

### 4.1 Participants and clinical findings

The outcome measurements and characteristics of examined cases are presented in Tables 1 and 2. Out of 40 patients, 53 posterior teeth were extracted. The test group included 22 patients with a total of 31 extracted teeth, while the control group included 18 patients, with a total of 22 extracted teeth. In some cases (*n* = 11), more than one tooth was extracted. In none of the cases a perforation of the Schneiderian membrane was observed during the extraction, the ARP, and the sinus lift. No infection in both groups, neither in the group with the exposed collagen membrane was observed.

**TABLE 1:** Outcome measurements of the number of sinus lifts and the mean bone‐height difference.

	Test group (*n* = 31)	Control group (*n* = 22)	*p*
Sinus lifts	4/31	7/22	0.168
Mean bone‐height difference (mm)	⌀ 0.14 ± 1.72	⌀ 0.17 ± 1.25	0.951

*Note: p*‐Values were obtained via *t*‐test for apparently normally distributed continuous variables (mean bone height differences) and Chi‐squared test for categorical variables (sinus lifts). The analysis for mean bone height differences between the groups excludes regions needing sinus floor elevation before implant placement, focusing on 15 teeth from the control group and 27 from the test group.

**TABLE 2:** Characteristics of examined cases.

Test group (*n* = 31)	Control group (*n* = 22)
Gender cases	
Male: 11/31	Male: 13/22
Female: 20/31	Female: 9/22
Age (mean ± SD)	
55.61 ± 13.43 years	51.09 ± 14.78 years
Location	
1st premolar: 8	1st premolar: 3
2nd premolar: 9	2nd premolar: 5
1st molar: 13	1st molar: 11
2nd molar: 1	2nd molar: 3
Mean pre‐operative bone height (mm)	
⌀ 11.78 ± 3.09	⌀ 11.13 ± 2.12
Mean post‐operative bone height (mm)	
⌀ 11.92 ± 2.79	⌀ 11,3 ± 2.17
Implant survival	
30/31	22/22
Implant length (mm)	
6 mm: 3	6 mm: 0
8 mm: 14	8 mm: 12
10 mm: 13	10 mm: 9
12 mm: 1	12 mm: 1
Implant diameter (mm)	
3.5 mm: 1	3.5 mm: 0
3.75 mm: 15	3.75 mm: 11
4.0 mm: 4	4.0 mm: 5
4.5 mm: 11	4.5 mm: 6

*Note*: Data are presented as mean ± standard deviation (SD).

### 4.2 Sinus lift augmentation

**FIGURE 14:**
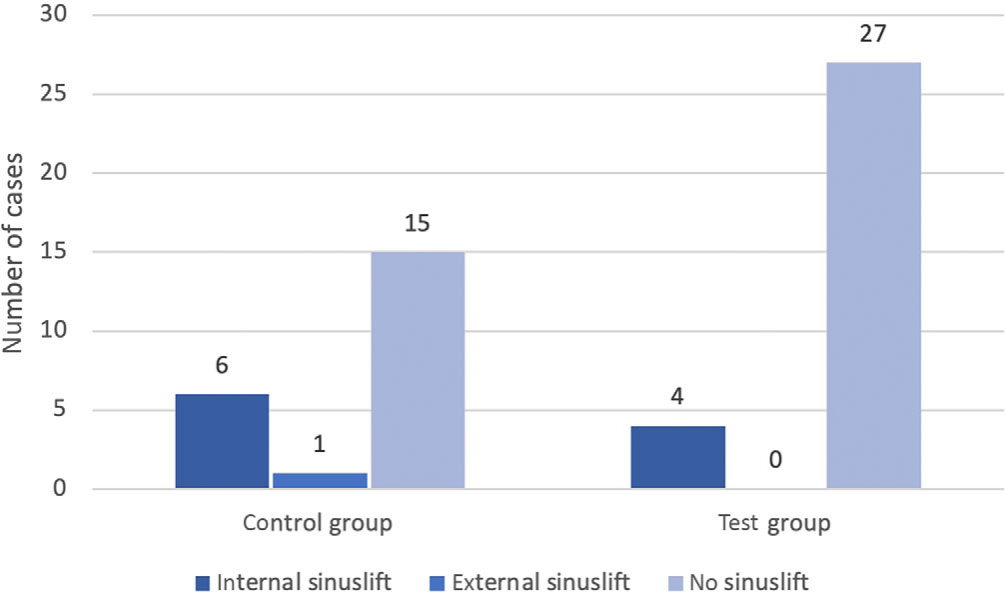
Number of cases for sinus lift.

Figure 14 visualizes the number of cases where sinus lifts were needed. A total of 11 sinus floor elevations were performed: 10 internal and one external.

In the control group, seven sinus floor elevations were performed, while only four were performed in the study group. The only external sinus floor elevation was performed in the control group.

Less sinus lifts were performed in the test group (odds ratio 0.32; 95% CI: 0.08, 1.26). However, the Alveolar Ridge Preservation using bovine bone substitute material was thus unable to prevent the need for sinus floor elevation, as no statistically significant differences in the number of sinus floor elevations performed could be determined between the control group and the test group (*p* = 0.168, Table 1).

### 4.3 Implant survival

All the surgeries related to implant insertion had an uneventful healing without any complication. All inserted implants in the test group as well as in the control group were as expected osseointegrated and received, after an average healing period of 5 months the definitive crowns, with one exception in the test group where one implant was early lost, because of failed osseointegration.

The survival rate of the implants in the control group was 100%, while it was 96.77% in the test group (Table 2).

According to the criteria of Buser and colleagues,^15^ a success rate of 100% was determined for the implants in the control group. In the test group, however, the success rate was 96.77% (Table 2).

### 4.4 Radiographic analysis

**FIGURE 15:**
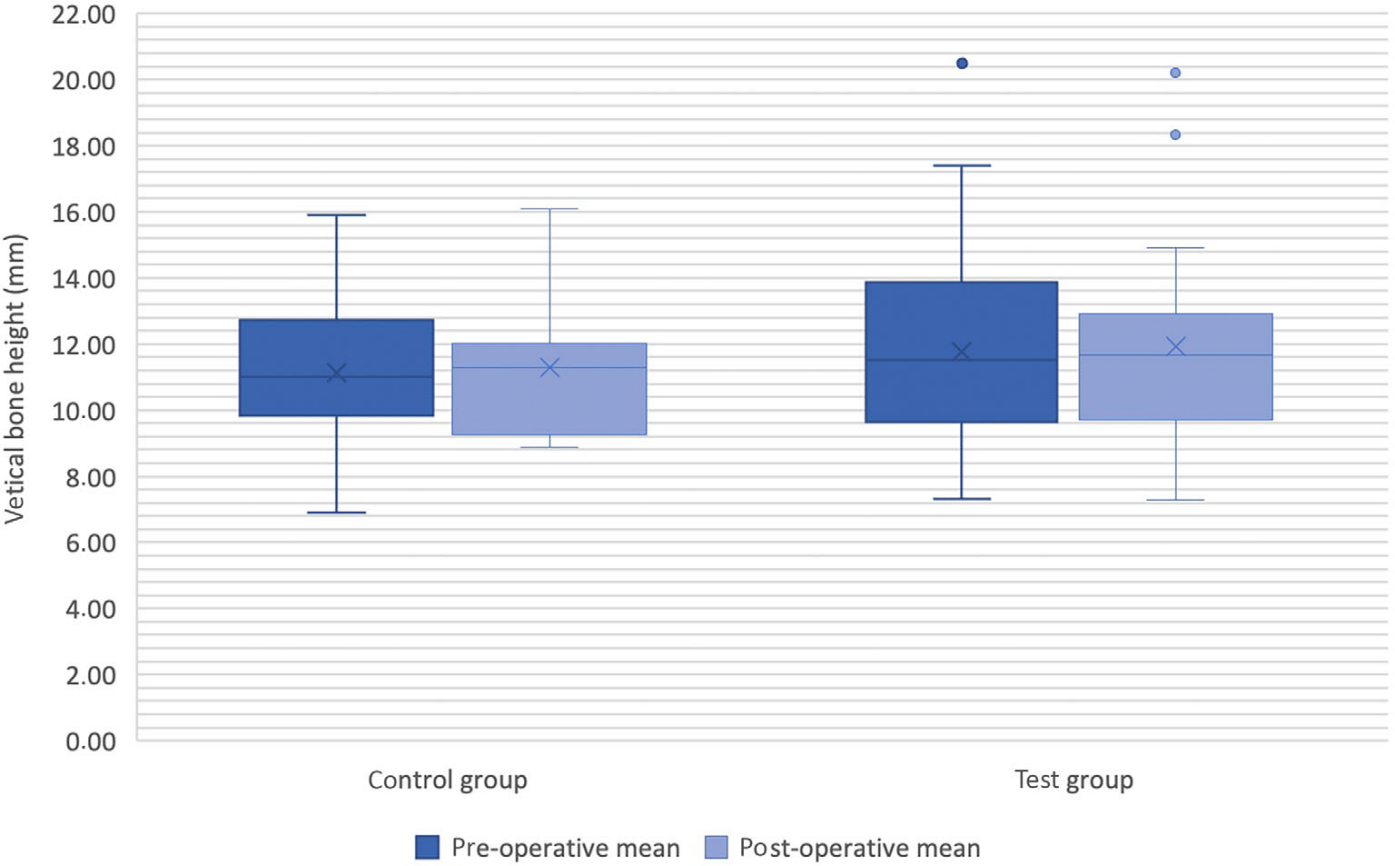
Vertical bone height in both group.

In the control group, the mean value of the radiographically measured bone height in (mesial and distal) was 11.13 ± 2.12 mm preoperatively before tooth extraction, while it was 11.3 ± 2.17 mm postoperatively after implant placement. In contrast, the mean value in the test group was 11.78 ± 3.09 mm preoperatively and 11.92 ± 2.79 mm postoperatively (Table 2 and Figure 15). The analysis does not include the regions which required a sinus floor elevation prior to implant placement. Thus, the analysis refers to 15 teeth from the control group and 27 teeth from the test group.

A *t*‐test demonstrated that there was no significant difference in the changes in pre‐ and postoperative bone height between the control group and the test group (95% CI: −1.01, 1.08; *p* = 0.951, Table 1).

## 5 DISCUSSION

After tooth extraction, the bundle bone that lines the periodontal ligament around the tooth and anchors the sharpey fibers in the bone is usually resorbed^6^ and replaced with woven bone, causing a vertical and horizontal reduction of the alveolar crest.^7^ Furthermore, loss of the vertical dimension especially in the posterior maxilla is the result of a combination of the resorption of the bundle bone and sinus pneumatization.

In order to achieve the best possible aesthetic outcome through functional prosthetic rehabilitation supported by osseointegrated implants, it may be crucial to preserve the alveolar ridge dimensions and various studies have already confirmed the effectiveness of ARP in reducing the bone loss after tooth removal.^8^, ^10^, ^16^ Even though the ARP cannot not fully prevent the resorption of the alveolar bone, studies have shown, that the volume of the alveolus is significantly less reduced compared to spontaneous healing.^17^

The present clinical trial investigated the clinical benefit of Alveolar Ridge Preservation by reducing the resorption of the bundle bone and for acting against the pneumatization in comparison to spontaneous healing without any alveolar filling material. The idea was if with the ARP the need of a sinus floor augmentation can be prevented. The results of this controlled prospective study indicated that Alveolar Ridge Preservation in the posterior maxilla, reduced need for sinus augmentation procedures, however not statistically relevant (*p* = 0.168, Table 1).

After tooth extraction, the socket in the test group was filled with bovine bone graft substitute and covered with a resorbable porcine membrane Although no primary wound closure was attempted and the membranes were exposed in oral cavity after suturing, new soft tissue formation was observed without any signs of inflammation in all cases. These findings were confirmed by recent studies which showed that exposed biodegradable membranes do not negatively affect bone regeneration of the fresh extraction socket and soft tissue formation.^18^ Furthermore, in the present study, new soft tissue formation without signs of inflammation or immunological reaction was observed up to the follow‐up period of 6 months. These data are confirmed by a systematic review by Wang and Lang,^19^ which showed that primary wound closure had no positive effect on the preservation of the alveolar ridge.

Although it is not a standard of precaution, all patients received antibiotics. It is commonly reported in the literature and seems to be indicated to prescribe antibiotics for extractions and dental implant placement when biomaterials are used to prevent complications.^20^ With the intention of limiting the experimental bias antibiotics were also given to the control group. During the follow‐up period, no postsurgical wound healing complications were observed.

At surgical re‐entry after 6 months, all alveolar sockets were filled with hard tissue in both groups. Titanium implants were placed without any additional augmentations except for four patients in the test group (12.9%) and seven (31.82%) in the control group who received further sinus lift augmentation. Of these 11 patients who required further augmentation procedures in the form of a sinus lift, 10 were internal sinus lifts and 1 was an external sinus lift. The external sinus lift had to be performed in the control group (Figure 14).

Even though sinus augmentation procedures are an effective and successful treatment option to increase the alveolar bone height and thus enable to place implants in the posterior maxilla,^21^, ^22^ they have a noticeable risk of complications such as perforation of the sinus membrane or infection.^23^ The present study demonstrated that ARP indicated to lower the need for sinus lift augmentations, however not statistically different on a *p* value of 0.05 (*p* = 0.168, Table 1). This occurrence is in line with findings reported by Lee and colleagues.^24^ The authors showed that 15.8% of patients who did not receive ARP required an additional sinus lift. In comparison, only 6.5% of patients in the group with ARP required an additional sinus lift,^24^ however not statistically relevant.

These findings are also presented with the previous study by Barone and colleagues^25^ who reported that only 7% of sites in the test group who received ARP with corticocancellous porcine bone, and a collagen membrane required an additional bone augmentation compared to 42% in the control group who did not receive any grafting material. However, it should be noted that in some of the extracted teeth the alveolar walls were not intact and patients who had suffered a traumatic extraction were included in the study population. In addition, smoking was not an exclusion criterion in this study. Patients who smoked less than 10 cigarettes per day were only asked to stop smoking before and after surgery. This could not be verified. In a prospective study conducted by Saldanha and colleagues, it was shown that smoking can negatively influence dimensional changes and the healing process of the extraction sockets.^26^ Moreover implant survival can also be significantly negatively influenced by smoking.^27^ In a study conducted by Daftari and colleagues,^28^ it was also shown that smoking inhibits revascularization of bone grafts, largely due to its vasoconstrictor effect on the arteries. Therefore, non‐smokers were selected in the present study to avoid a major factor that could interfere with or disrupt normal bone graft healing.

In the present study, no vertical bone resorption was recorded in test and in control sites after 6‐months follow‐up (Figure 15). Furthermore, there was no statistically significant distinction observed in the vertical alterations of the ridge between the two groups (95% CI: −1.01, 1.08; *p* = 0.951). The test group and the control group almost maintained the vertical bone level without significant change (test group: 11.78 ± 3.09 mm preoperatively before tooth extraction and 11.92 ± 2.79 mm postoperatively after implant placement, control group: 11.13 ± 2.12 mm preoperatively, while it was 11.3 ± 2.17 mm postoperatively, Figure 15). These findings are not in agreement with the results from Cha and colleagues who reported a significantly larger bone height in the test group who received ARP using collagenated bovine bone mineral and a resorbable collagen membrane in comparison to the control group.^8^ Furthermore, a meta‐analysis by Willenbacher and colleagues found that ARP preserved an average of approximately 1.31–1.54 mm more bone width and 0.91–1.12 mm more bone height compared to spontaneous healing.^29^ Although the study underlined that ARP cannot completely stop alveolar ridge resorption, it can limit it more effectively though the use of bone substitute materials.^29^

The total survival rate of the implants placed was 100% in the control group and 96.77% in the test group. One implant was lost in the test group (length: 8 mm, diameter: 3.5 mm) during the study. Implants inserted in augmented areas with ARP showed a high success rate, despite direct contact with bovine bone substitute. Similar results were obtained in the study conducted by Barone and colleagues.^30^ This study compared the success rates for Implants placed in augmented versus non‐augmented extraction sockets.^30^ At the 3‐year follow‐up, the combined success rate of the implants for both groups was recorded at 95%.^30^ The analysis of the implant success rates for both groups, considering the length and diameter of the implants, can be summarized as follows: The overall survival rate of all implants in the test and control group was 98.12%. The survival rate of the implants with a length of 6 and 10 mm was 100% after 12 months. Due to the loss of one implant in the group of 8 mm implants, the survival rate at this site was 96.3%. Other studies also confirm the high implant survival rates of different implant lengths placed after ARP^30^, ^31^, ^32^ and spontaneous healing.^33^ The survival rate of the implants with diameters of 3.75, 4, and 4.5 mm in both groups was 100%. In contrast, the survival rate for the group of implants with a diameter of 3.5 mm was 0%. However, this is because only one implant with such a diameter was implanted. Here too, the overall survival rate was 98.12%. These survival rates are consistent with those found in the literature. In 2012, Al‐Nawas and colleagues^34^ showed that the implant diameter of 3.5 or 4 mm had no significant influence on the survival rate of the implants after an average duration of 108 months.^34^ This finding was also confirmed by other studies.^35^, ^36^

When asked in which cases alveolar ridge preservation has a benefit for adequate preservation of hard and soft tissue, the biotypes of soft tissue described by Olsson and colleagues^37^, ^38^ could be of great importance for prognosis. A thin soft tissue, especially in the maxilla can be associated with thin vestibular bone, which is rapidly resorbed as a consequence of tooth extraction.^39^ In a thick biotype, on the other hand, resorption after tooth extraction may play a rather subordinate role due to the thick vestibular bone. Thus, the significance of ARP could be significantly higher in a thin gingival biotype than in a thick biotype. However, a systematic review and meta‐analysis by Avila‐Ortiz and colleagues^10^ found that ridge regions with a buccal bone thickness of more than 1.0 mm exhibited more favorable ridge preservation outcomes than regions with a thinner buccal wall. This is in line with the results of Nevins and colleagues^17^ where it was shown that although ARP cannot preserve the buccal bone wall in all cases, the volume of the alveolus is significantly less reduced compared to spontaneous healing.

This research acknowledges several limitations that must be taken into consideration. It is important to remark that one of the potential limitations of this study is the small sample size in both groups. These results reveal the need for further research involving a larger patient population to support and substantiate our findings, as the limited number of patients in our study prevents drawing definitive, meaningful conclusions. The necessity for further investigation is especially relevant considering the trends observed in our results, which, although suggestive, require more extensive data for final confirmation. Furthermore, the discrepancy in outcomes between our study and others may be due to this difference in patient sample size. Therefore, studies involving a larger number of patients may not only confirm the trends we observed, but also provide insight into the comparative results seen in different studies.

Another limitation of the current study is the absence of an analysis of horizontal bone loss. To comprehensively evaluate changes in horizontal bone width, 3‐dimensional imaging, such as beam‐computed tomography (CBCT), is essential. Due to ethical considerations and the need to minimize exposure to radiation, CBCT scans were only performed prior to implant placement for the purpose of implant planning. Therefore, additional 3‐dimensional imaging would be necessary to allow an in‐depth analysis of horizontal bone loss. The lack of these subsequent imaging sessions hinders our ability to evaluate bone changes in the horizontal dimension over time.

## 6 CONCLUSION

Within the limits of this study, the use of bovine bone substitute and a porcine resorbable membrane after tooth extraction in the posterior maxilla seems to reduce the need for sinus augmentation compared to spontaneous healing, although the difference was not statistically significant. Nevertheless, the ARP in the test group made external sinus floor elevation unnecessary compared to the control group. The change in radiographically measured bone height pre‐ and postoperatively showed no significant difference between the two groups. Further investigation with a larger sample size is recommended to confirm these findings.

## AUTHOR CONTRIBUTIONS

Khoury, Elias Jean‐Jacques—Concept/Design, Data collection, Drafting article. Sagheb, Keyvan—Concept/Design, Drafting article, Critical revision of article, Approval of article, Data collection, Surgical procedures. Al‐Nawas, Bilal—Concept/Design, Drafting article, Critical revision of article, Approval of article, Data collection, Surgical procedures. König, Jochem—Concept/Design, Data analysis/interpretation. Schiegnitz, Eik—Concept/Design, Drafting article, Critical revision of article, Approval of article, Data collection, Surgical procedures.

## FUNDING INFORMATION

Funding secured by Straumann, Switzerland.

## CONFLICT OF INTEREST STATEMENT

Elias Jean‐Jacques Khoury declares that he has no conflict of interest. Keyvan Sagheb reports lectures, personal fees and/or grants from Dentsply, Geistlich, and Straumann outside the submitted work. Bilal Al‐Nawas reports lectures, personal fees and/or grants from Camlog, Dentsply, Geistlich, Medartis, Straumann and Zimmer outside the submitted work. Jochem König declares that he has no conflict of interest. Eik Schiegnitz reports lectures, personal fees and/or grants from Dentsply, Geistlich, Medartis, Septodont and Straumann outside the submitted work.

## Data Availability

Research data are not shared.
